# The impact of type 2 diabetes on left ventricular function in hypertensive patients: a three-dimensional speckle-tracking imaging study

**DOI:** 10.3389/fcvm.2024.1453809

**Published:** 2025-01-24

**Authors:** Yang Qingmei, Chen Xiaoyan, Fang Jianxiu

**Affiliations:** Department of Ultrasonography, Third Hospital of Shanxi Medical University, Shanxi Bethune Hospital, Shanxi Academy of Medical Sciences, Tongji Shanxi Hospital, Taiyuan, China

**Keywords:** hypertension, type 2 diabetes, left ventricle, train, three-dimensional speckle-tracking imaging, systolic dysfunction

## Abstract

**Objectives:**

The study aimed to evaluate the impact of the co-occurrence of hypertension and type 2 diabetes mellitus (T2DM) on the deterioration of left ventricular (LV) structure and function using three-dimensional speckle-tracking imaging (3D-STI), compared to patients with only hypertension.

**Methods:**

Data from 272 hypertensive patients, including 85 with T2DM and 187 without, along with 45 normal controls, were analyzed. Participant characteristics were assessed before and after propensity score matching (PSM). 3D-STI-derived parameters, including LV function and global strain parameters, were compared among controls and different patient groups. Multivariable linear regression analyses were conducted to determine the impacts of T2DM on LV function and global strain. Additionally, linear mixed-effects regression models were used to evaluate the associations between 3D-STI-derived parameters and T2DM over time in hypertensive patients.

**Results:**

Significant increases in the E/e' ratio and declines in the LV global radial strain (GRS) were observed across the control group, HTN (T2DM-) group, and HTN (T2DM+) group. After adjusting for various factors using PSM analysis, LV global circumferential strain (GCS) and global area strain (GAS) were also found to be significantly decreased in the HTN (T2DM+) group compared to the HTN (T2DM-) group. Multivariable regression analyses, accounting for various covariates, indicated that T2DM was independently linked to LV strains (LV GAS: *β* = 0.95, 95% CI: 0.90–1.00, *p* = 0.029; LV GRS: *β* = 1.03, 95% CI: 1.01–1.06, *p* = 0.014) in hypertensive patients. Furthermore, linear mixed-model analysis revealed that LV GCS (*β* = 1.20, 95% CI: 0.38–2.01, *p* = 0.004) and GRS (*β* = −2.82, 95% CI: −4.97–0.68, *p* = 0.010) deteriorated over the 12-month period.

**Conclusions:**

T2DM exacerbates the decline in LV global and regional strains in patients with hypertension, and 3D-STI may be a valuable tool for detecting these asymptomatic preclinical abnormalities.

## Introduction

Cardiovascular disease is the leading cause of mortality among patients with hypertension (1). Hypertension and T2DM often coexist due to shared lifestyle factors and pathophysiological mechanisms (2). Studies show that about half of individuals with T2DM also have hypertension, with an even higher prevalence among hospitalized patients (3). Both conditions can lead to significant cardiac structural remodeling and dysfunction (4). Hypertension causes adverse changes in cardiac structure and function, such as LV hypertrophy, due to increased afterload (5). Diabetes is associated with a higher risk of heart failure and specific alterations in LV structure and function (6). However, many studies on the relationship between hypertension, diabetes, and cardiovascular disease (CVD) outcomes have not conducted stratified analyses to determine if the associations of diabetes with changes in LV structure and function are independent of hypertension. Therefore, it has been difficult to separate the independent effects of diabetes on cardiac structure and function from those of comorbid hypertension.

Noninvasive imaging techniques have gained significant interest for studying functional changes in the LV across a range of diseases. Evaluation of myocardial contractility using echocardiography has traditionally been performed through volume-based assessment of ejection fraction and estimation of myocardial thickening (7). However, these conventional methods are sensitive to loading conditions and can be variable due to assumptions about geometric modeling and errors from foreshortened echocardiographic views (8). Over the past decade, advancements in cardiac imaging have introduced new echocardiographic techniques, such as two-dimensional (2D) speckle-tracking and 3D echocardiography (9). These techniques enable more accurate quantification of ventricular function. Compared to 2D speckle-tracking imaging (2D-STI), 3D-STI offers higher accuracy and reproducibility in evaluating LV volume and function. It provides a more comprehensive assessment of myocardial motion without out-of-plane speckle loss, offers strain information that is closer to reality, and more reliably evaluates LV myocardial function (10–12). Current research on 3D-STI predominantly focuses on conditions like coronary heart disease, cardiomyopathy, valvular heart disease, and other cardiac disorders (10, 13, 14). This advanced technique holds significant value in accurately evaluating myocardial function, aiding in differential diagnosis, risk stratification, and predicting adverse events.

This study aimed to assess the impact of T2DM on LV structural and functional changes in individuals with hypertension using three-dimensional speckle-tracking imaging (3D-STI). Additionally, the study tracked changes in both risk factors and cardiovascular indices in hypertensive patients over a 12-month period.

## Materials and methods

### Study population

**Figure 1:**
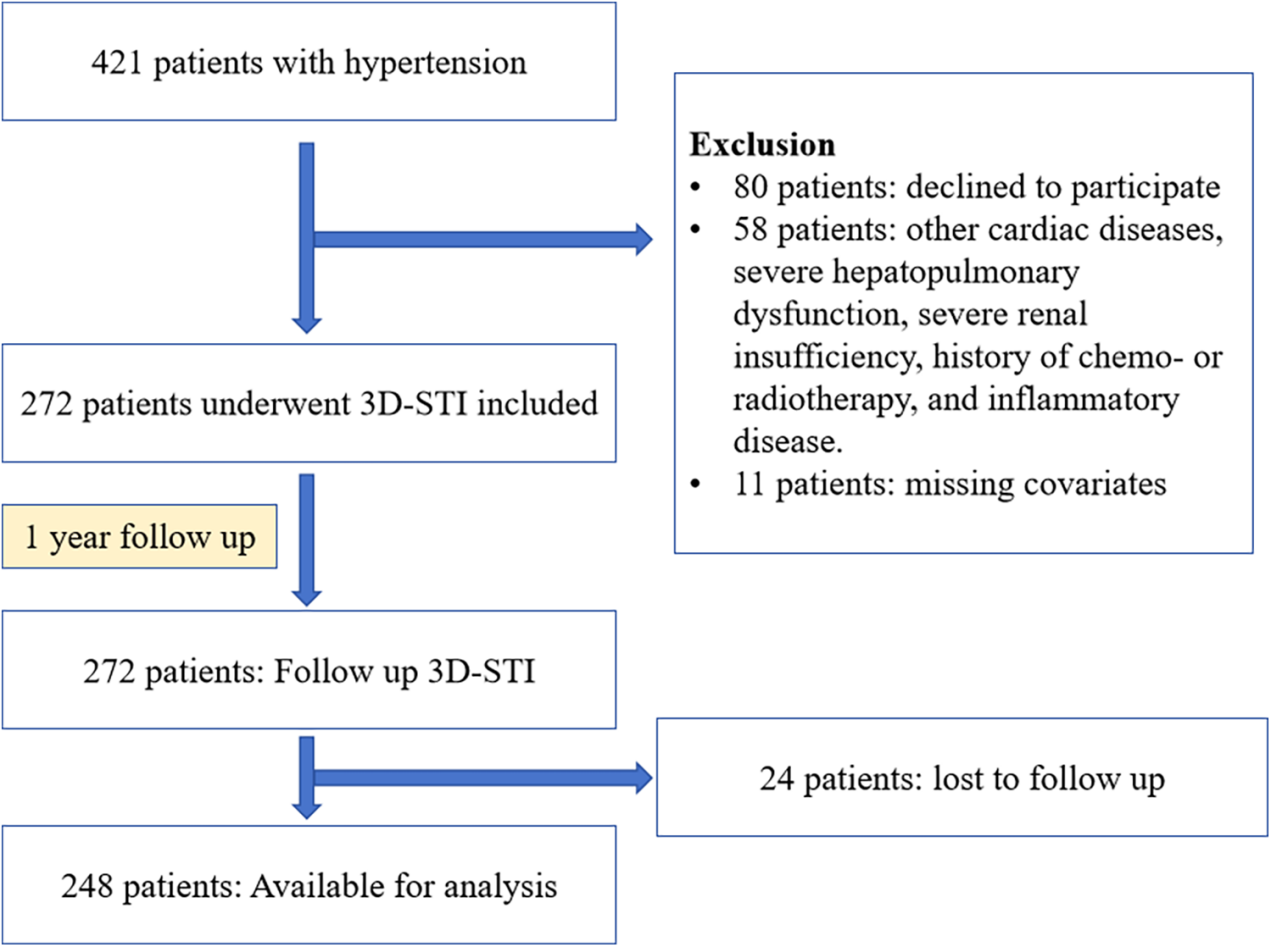
Flowchart of participants throughout the study.

This prospective study took place at Shanxi Baiqiuen Hospital in Shanxi, China, from November 2020 to April 2022. Figure 1 illustrates the detailed steps of the entire screening process. A total of 421 adult patients with essential hypertension who underwent real-time three-dimensional standard echocardiographic assessment at the hospital were enrolled and categorized into two groups: those with T2DM [HTN (T2DM+)] and those without [HTN (T2DM-)]. Hypertension was defined as a systolic blood pressure (SBP) ≥ 140 mmHg and/or a diastolic blood pressure (DBP) ≥ 90 mmHg at rest, measured on multiple occasions, or the use of antihypertensive medications. The diagnosis of T2DM followed the guidelines of the American Diabetes Association, which includes HbA1c levels ≥6.5% or fasting plasma glucose ≥126 mg/dl.

Exclusion criteria were the following: history of coronary heart disease or known cardiopathy (including myocardial infarction, percutaneous coronary intervention, or coronary artery bypass grafting), previous documented diagnosis of cardiac damage or heat failure, left or right ventricular ejection fraction <50%, atrial fibrillation, moderate to severe valvular disease, severe renal insufficiency (eGFR < 30 ml/1.73 mm^2^) or dialysis, history of chemo- or radiotherapy, congenital heart disease, inflammatory diseases (including rheumatoid arthritis, systemic lupus erythematosus, inflammatory bowel diseases, vasculitis, or any condition associated with acute or chronic systemic inflammation), and myocarditis. Patients missing covariates were also excluded. Ultimately, 272 patients met the eligibility criteria for this study. 45 healthy individuals with no history of impaired glucose tolerance, electrocardiogram (ECG) abnormalities, symptoms of cardiovascular disease or cardiovascular abnormalities detected using 3D echocardiography were included as the control group.

In addition to baseline echocardiography, the hypertension group underwent longitudinal examinations at 12 months to evaluate changes in cardiac indices over time. To enhance retention, participants received regular reminders via phone and email, and small incentives were provided at each follow-up visit. Comprehensive clinical assessments, including physical exams and echocardiography, were conducted, with echocardiograms analyzed by blinded cardiologists using standardized equipment. Linear mixed-effects models were employed for data analysis, and missing data were managed through multiple imputation to ensure robustness in the final results. This study received approval from the Biomedical Research Ethics Committee of Shanxi Baiqiuen Hospital and was conducted in accordance with the Declaration of Helsinki.

### Standard echo-Doppler examination

Conventional echocardiography was performed to analyze a range of cardiac parameters. Interventricular septal thickness (IVSd) was measured. Pulsed wave Doppler ultrasound was used to collect transmitral inflow diastolic velocities (E and A). Tissue Doppler imaging was employed to measure mitral annular diastolic velocities (e' and A') at both the septal and lateral mitral annulus (7). The average of these two measurements for e' was used for subsequent analysis. The ratios of E/A, e'/a', and E/e' were then calculated as key indices of diastolic function and left ventricular filling pressure.

### Three-dimensional speckle-tracking imaging

Transthoracic echocardiographic examinations were conducted by a skilled echocardiographer using the X5-1 transducer and either an EPIQ 7G or EPIQ Elite ultrasound machine (Philips, Bothell, WA, USA). All measurements adhered to the current guidelines of the American Society of Echocardiography. The examination protocol involved assessing the left ventricle in the standard four-chamber apical view. To achieve a high frame rate, the field of view was optimized by adjusting the sector width and depth and utilizing zoom when necessary.

Speckle-tracking echocardiography was employed to evaluate the global longitudinal strain of the left ventricle, with measurements taken at a rate of 50–70 frames per second. The clips were saved at the original frame rate to an external hard drive and later analyzed offline using QLAB Advanced Quantification software (Philips). The software generated the LV volume-time curve, left ventricular ejection fraction (LVEF), and global and regional strain values in various directions. The 3D routine data and strain parameters included LVEF, left ventricular mass (LVM), left ventricular mass index (LVMi), and left ventricular end-diastolic mass (LVEDMass). Global longitudinal strain (GLS), global circumferential strain (GCS), global area strain (GAS), and global radial strain (GRS) values were determined as the weighted averages of their respective peak strain values using the software.

### Statistical analysis

Statistical analysis was conducted using SPSS version 27.0 software. Categorical data are presented as frequencies (percentages) and were compared using the chi-squared test. The normality of continuous variables was assessed using the Shapiro-Wilk test. Due to the non-normal distribution of variables, data were described using interquartile ranges. The Kruskal-Wallis test was utilized to compare baseline clinical characteristics and cardiac measurements. Given the non-randomized study design, we conducted PS matching analysis to compare the cardiac 3D-STI-derived parameters between [HTN (T2DM-)] and [HTN (T2DM+)] patients. Spearman's correlation coefficient was employed to analyze the relationships between 3D-STI-derived LV function and ventricular volumetrics, LV global strains, and regional strains in hypertensive patients. Multiple linear regression analyses were conducted to investigate the association between T2DM and various LV global strains, adjusting for BMI, age, sex, duration of hypertension, systolic blood pressure (SBP), diastolic blood pressure (DBP), heart rate, and use of ACEI/ARB. We matched each patients with diabetes with those without diabetes at a 1:1 ratio using the optimal method, with a caliper width equal to 0.3 of the standard deviation of the logit PS. The balance of baseline features was examined, and a standardized mean difference < 0.1 indicated a negligible difference. Linear mixed-effects models with two random effects (random intercept and random slope) and an unstructured variance-covariance matrix, were used to assess the relationships between 3D-STI-derived parameters and T2DM in the patients with hypertension over time. Statistical significance was set at *P* < 0.05, and all tests were two-tailed.

## Results

### Demographic and clinical characteristics of study participants at baseline

Table 1 presents the baseline clinical characteristics of the participants. Both patient groups exhibited significantly higher age, BMI, heart rate, SBP, and DBP compared to the control group (all *p* < 0.01). The PS matching analysis included 272 patients who had hypertension. After 1:1 PS matching, 170 patients were included in the final analysis. The demographic and clinical parameters at baseline were well balanced, with a standardized mean difference < 0.1 (Table 1). In the HTN (T2DM+) group, fasting blood glucose and plasma triglycerides (*p* = 0.000) levels were significantly higher than in the HTN (T2DM-) group and controls in both the unmatched and PS-matched cohorts (all *p* < 0.01). Additionally, LDL levels in the HTN (T2DM+) group were significantly lower than those in the HTN (T2DM-) (*p* = 0.035) and control groups (*p* < 0.01).

**Table 1:** Baseline characteristics of the study population.

Parameter	All patients (*n* = 317)			*p* value	PS-matched pairs (*n* = 170)		*p* value
	Controls (*n* = 45)	HTN (T2DM-) (*n* = 187)	HTN (T2DM+) (*n* = 85)		HTN (T2DM-) (*n* = 85)	HTN (T2DM+) (*n* = 85)	
Demographics							
Female, *n* (%)	21 (46.7)	102 (54.5)	51 (60.0)	0.228	47 (55.3)	51 (60.0)	0.535
Age (year)	51.0 (45.5, 54.0)	65.0 (62.0, 69.0)*	65.0 (63.0, 68.0)*	0.000	64.0 (62.0, 67.5)	65.0 (63.0, 68.0)	0.299
BMI (kg/m^2^)	24.0 (22.9, 25.7)	25.8 (23.9, 27.8)*	26.0 (24.0, 28.3)*	<0.001	26.2 (24.2, 27.8)	26.0 (24.0, 28.3)	0.940
BSA (m^2^)	1.69 (1.65, 1.77)	1.70 (1.61, 1.83)	1.73 (1.58, 1.83)	0.886	1.73 (1.64, 1.83)	1.73 (1.58, 1.83)	0.764
Duration of hypertension (year)	0	14.5 (8.2, 21.4)	11.6 (6.7, 18.4)	0.015	13.0 (7.9, 20.4)	11.6 (6.7, 18.4)	0.422
Duration of diabetes (year)	0	0	1.2 (0.7, 1.6)	NA	0	1.2 (0.7, 1.6)	NA
Laboratory data							
Fasting blood glucose (mmol/L)	4.67 (3.76, 5.41)	5.34 (4.76, 5.70)*	6.97 (6.46, 8.07)*^,^^#^	0.000	5.33 (4.91, 5.69)	6.97 (6.46, 8.07)^#^	0.000
HbA1c (%)	5.42 (5.09, 5.94)	6.22 (6.03, 6.57)	7.32 (7.14, 8.43)	0.000	6.20 (6.10, 6.64)	7.32 (7.14, 8.43)	0.000
Triglycerides (mmol/L)	1.18 (1.05, 1.37)	1.50 (1.13, 2.14)*	1.84 (1.31, 2.47)*^,^^#^	0.000	1.70 (1.13, 2.12)	1.84 (1.31, 2.47)^#^	0.023
Total cholesterol (mmol/L)	4.18 (3.71, 4.50)	4.44 (3.92, 4.94)*	4.43 (3.96, 5.10)*	0.000	4.43 (3.89, 4.89)	4.43 (3.96, 5.10)	0.506
HDL (mmol/L)	1.01 (0.86, 1.51)	2.61 (2.11, 3.08)*	2.54 (1.98, 3.11)*	0.000	2.63 (2.12, 3.06)	2.54 (1.98, 3.11)	0.923
LDL (mmol/L)	2.80 (2.63, 3.03)	1.38 (1.22, 1.57)*	1.36 (1.19, 1.54)*^,^^#^	0.000	1.42 (1.25, 1.63)	1.36 (1.19, 1.54)^#^	0.035
Hemodynamic variables							
Heart rate (beats/min)	67.0 (62.0, 71.5)	74.0 (68.0, 82.0)*	75.0 (66.8, 83.5)*	<0.001	74.0 (67.5, 81.5)	75.0 (66.8, 83.5)	0.689
SBP (mmHg)	118.0 (112.0, 126.5)	170.0 (160.0, 174.0)*	169.0 (160.0, 180.0)*	0.000	170.0 (160.0, 176.5)	169.0 (160.0, 180.0)	0.912
DBP (mmHg)	78.0 (72.5, 82.0)	100.0 (90.0, 110.0)*	100.0 (90.0, 105.3)*	0.000	100.0 (90.0, 110.0)	100.0 (90.0, 105.3)	0.718
Antihypertensive medications							
ACEI/ARB, *n* (%)	0	88 (47.1)	37 (43.6)	0.073	44 (51.8)	37 (43.6)	0.309
Calcium channel blocker, *n* (%)	0	163 (87.1)	75 (88.2)	0.591	78 (91.8)	75 (88.2)	0.305
Diuretic, *n* (%)	0	21 (11.2)	9 (10.6)	0.825	8 (9.4)	9 (10.6)	0.798
Hypoglycemic medications				NA			NA
Insulin	0	0	19 (22.4)		0	19 (22.4)	
Metformin	0	0	27 (31.8)		0	27 (31.8)	
Sulfonylureas	0	0	10 (11.8)		0	10 (11.8)	
*α*-Glucosidase inhibitors	0	0	33 (38.8)		0	33 (38.8)	

Values are interquartile ranges, numbers in the brackets are percentage.

BMI, body mass index; HDL, high-density lipoprotein cholesterol; LDL, low–density lipoprotein cholesterol; SBP, systolic blood pressure; DBP, diastolic blood pressure; ACEI, angiotensin converting enzyme inhibitor; ARB, angiotensin II receptor blocker.

* *p* < 0.05 vs. control group.

^#^ *p* < 0.05 vs. HTN (T2DM-) group.

### Comparison of the characteristics of the cardiac 3d-STI-derived parameters between HTN (T2dm-) and HTN (T2dm+) group

**Figure 2:**
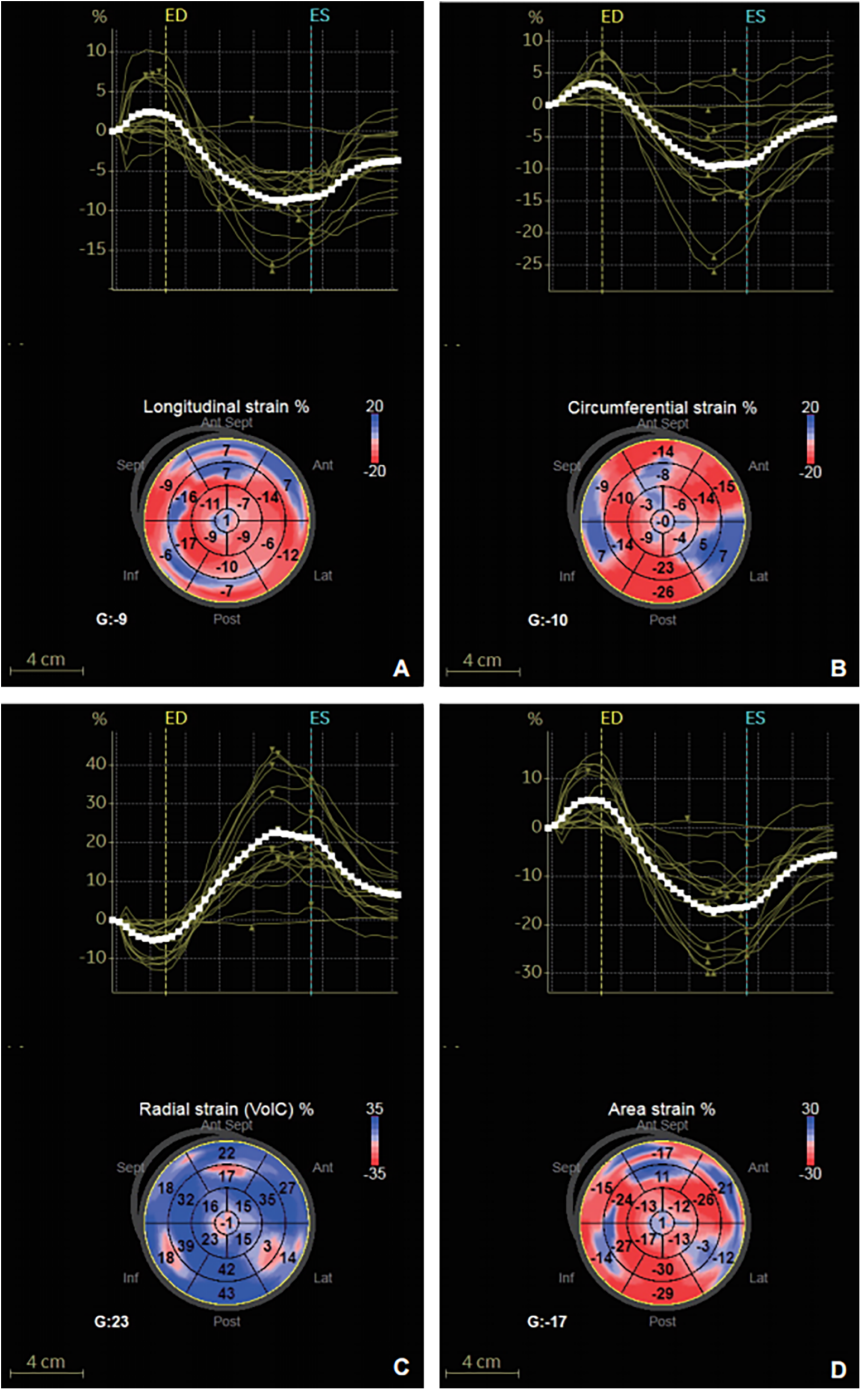
Strain-time curve and peak global systolic strain values were evaluated using the software in a 63-year-old hypertensive patients with T2DM. **(A)** GLS. **(B)** GCS. **(C)** GRS. **(D)** GAS.

Table 2 displays the cardiac 3D-STI parameters. Compared to controls, hypertensive patients exhibited higher left ventricular mass (LVM), interventricular septal thickness (*p* = 0.038), e' velocity (*p* = 0.000), and E/e' ratio (*p* < 0.001). The LVMi, E/A ratio (*p* = 0.000) were lower in the hypertension group compared to controls. LV GLS, GRS, GCS, and GAS decreased progressively from controls through the HTN (T2DM-) group to the HTN (T2DM+) group (all *p* < 0.01) (Figure 2).

**Table 2:** Comparison of 3D-STI-derived LV parameters among groups.

Parameter	All patients (*n* = 317)			*p* value	PS-matched Pairs (*n* = 170)		*p* value
	Controls (*n* = 45)	HTN(T2DM-) (*n* = 187)	HTN(T2DM+) (*n* = 85)		HTN(T2DM-) (*n* = 187)	HTN(T2DM+) (*n* = 85)	
LVM	136.43 (128.64, 141.61)	168.32 (147.23, 181.31)*	162.34 (142.11, 181.38)*	<0.001	163.85 (147.21, 180.62)	162.34 (142.11, 181.38)	0.828
LVMi (g/m^2^)	79.15 (73.98, 83.31)	96.39 (86.24, 107.63)*	92.33 (84.60, 103.10)*	<0.001	96.02 (84.13, 105.85)	92.33 (84.60, 103.10)	0.564
Interventricular septal thickness (mm)	10.0 (10.0, 10.0)	10.0 (10.0, 11.0)*	10.0 (9.0, 11.0)*	0.038	10.0 (10.0, 11.0)	10.0 (9.0, 11.0)	0.631
LVEF (%)	66.74 (65.14, 69.02)	67.0 (63.0, 70.0)	68.0 (64.0, 70.0)	0.725	67.0 (64.0, 70.5)	68.0 (64.0, 70.0)	0.856
E/A ratio	1.30 (1.07, 1.47)	0.76 (0.67, 0.85)*	0.75 (0.64, 0.84)*	0.000	0.77 (0.69, 0.85)	0.75 (0.64, 0.84)	0.157
e’ velocity (cm/s)	13.87 (12.29, 13.93)	8.0 (7.0, 9.0)*	8.0 (6.0, 9.0)*	<0.001	8.0 (7.0, 9.0)	8.0 (6.0, 9.0)	0.122
E/e’ ratio	6.44 (5.65, 7.58)	8.33 (7.00, 10.14)*	9.25 (7.80, 10.92)*^,^^#^	<0.001	8.25 (6.95, 9.94)	9.25 (7.80, 10.92)^#^	0.021
LV GLS (%)	−19.9 (−21.6, −18.1)	−10.0 (−13.0, −7.0)*	−9.0 (−12.0, −7.0)*	0.000	−11.0 (−13.0, −7.5)	−9.0 (−12.0, −7.0)	0.103
LV GCS (%)	−19.7 (−24.2, −14.4)	−13.0 (−15.0, −10.0)*	−11.0 (−15.0, −9.0)*	<0.001	−13.0 (−16.0, −10.0)	−11.0 (−15.0, −9.0)^#^	0.022
LV GAS (%)	−33.1 (−38.9, −28.3)	−21.0 (−24.0, −16.0)*	−18.0 (−23.0, −14.0)*	0.000	−21.0 (−24.5, −18.0)	−18.0 (−23.0, −14.0)^#^	0.008
LV GRS (%)	46.2 (42.0, 49.4)	31.0 (23.0, 38.0)*	26.0 (19.5, 35.5)*^,^^#^	0.000	31.0 (26.0, 38.5)	26.0 (19.5, 35.5)^#^	0.004
Spehericity index	0.38 (0.36, 0.41)	0.35 (0.31, 0.41)*	0.37 (0.30, 0.45)	0.091	0.36 (0.32, 0.43)	0.37 (0.30, 0.45)	0.924

LVM, left ventricular mass; LVMi, left ventricular mass index; LVEF, left ventricular ejection fraction; A, transmitral A velocity; E, transmitral E velocity; e’, e’ velocity of mitral annulus; GLS, global longitudinal strain; GCS, global circumferential strain; GAS, global area strain; GRS, global radial strain; EDmass, end-diastolic mass index.

* *p* < 0.05 vs. control group.

^#^ *p* < 0.05 vs. HTN (T2DM-) group.

The HTN (T2DM+) group demonstrated an increased E/e' ratio (*p* = 0.042) and a decreased LV GRS (*p* = 0.032) compared to the HTN (T2DM-) group. There were no significant differences in LVM, LVMi, interventricular septal thickness, LVEF) E/A ratio, e’ velocity, LVED mass, LV GLS, GCS, and GAS between the HTN (T2DM-) and HTN (T2DM+) groups (all *p* > 0.05). After PS matching, the duration of hypertension was comparable between HTN (T2DM-) and HTN (T2DM+) groups. In the PS-matched cohort, the HTN (T2DM+) group consistently exhibited higher E/e' ratio and lower LV GRS compared to the HTN (T2DM-) group. In addition, LV GCS (*p* = 0.022) and GAS (*p* = 0.008) were observed lower in the HTN (T2DM+) group compared to the HTN (T2DM-) group (see Table 2).

### Correlation analysis of cardiac 3D-STI derived parameters among hypertension patients

In patients with hypertension (Table 3), the E/A ratio was significantly correlated with LV GCS (*r* = −0.138, *p* = 0.022), GAS (*r* = −0.151, *p* = 0.013), and GRS (*r* = 0.164, *p* = 0.007), but not GLS (*r* = −0.084, *p* = 0.166). Additionally, the E/e' ratio showed the strongest association with GCS (*r* = −0.130, *p* = 0.033) (see Table 3).

**Table 3:** Correlation between 3D-derived LV components in the patients with hypertension (r coefficient and significance).

	GLS (*p*-value)	GCS (*p*-value)	GAS (*p*-value)	GRS (*p*-value)
BMI	0.125 (0.039)	0.111 (0.067)	0.141 (0.020)	−0.125 (0.040)
SBP	−0.055 (0.370)	−0.004 (0.952)	−0.029 (0.639)	0.015 (0.808)
DBP	−0.024 (0.697)	0.015 (0.811)	−0.023 (−0.700)	0.024 (0.693)
LVEF	0.100 (0.100)	−0.089 (0.142)	0.018 (0.766)	−0.024 (0.697)
LVM	0.035 (0.561)	−0.057 (0.347)	0.010 (0.868)	0.001 (0.988)
LVMi	−0.088 (0.146)	−0.074 (0.224)	−0.065 (0.288)	0.067 (0.274)
E/A ratio	−0.084 (0.166)	−0.138 (0.022)	−0.151 (0.013)	0.164 (0.007)
e’ velocity	−0.111 (0.069)	0.000 (0.998)	−0.075 (0.218)	0.105 (0.084)
E/e’ ratio	−0.037 (0.542)	−0.130 (0.033)	−0.094 (0.122)	0.073 (0.234)

BMI, body mass index; SBP, systolic blood pressure; DBP, diastolic blood pressure; LVEF, left ventricular ejection fraction; LVM, left ventricular mass; LVMi, left ventricular mass index; A, transmitral A velocity; E, transmitral E velocity; e’, e’ velocity of mitral annulus.

### Multivariate regression analyses of T2DM with LV strains in patients with hypertension

The univariable analysis revealed that age, BMI, sex, SBP, DBP, and heart rate were all significantly associated with LV strains (all *p* < 0.05; see Table 4). In the multivariable analysis, increases in age, BMI, sex, SBP, DBP, and heart rate remained independent determinants of reduced LV strains among all patients (all *p* < 0.05). Variables with a *p*-value < 0.10 in the univariable model were included in the multivariable model to evaluate the impact of T2DM on LV strain in patients with hypertension. After adjusting for BMI, age, sex, duration of hypertension, SBP, DBP, and heart rate, multivariable regression analyses of the patients with hypertension showed that T2DM was independently associated with LV GAS (*β* = 0.949, 95% CI: 0.905-0.995, *p* = 0.031) and GRS (*β* = 1.033, 95% CI: 1.006–1.060, *p* = 0.014), but not with LV GLS or GCS. In addition, after adjusting for the above covariates and ACEI/ARB use, T2DM was independently correlated with LV GAS (*β* = 0.949, 95%CI: 0.904–0.995, *p* = 0.029) and GRS (*β* = 1.033, 95%CI: 1.007–1.061, *p* = 0.014) (see Table 5).

**Table 4:** Determinants of LV strains in patients with hypertension.

	GLS				GCS				GAS				GRS			
	Univariable	*p*-value	Multivariable	*p*-value	Univariable	*p*-value	Multivariable	*p*-value	Univariable	*p*-value	Multivariable	*p*-value	Univariable	*p*-value	Multivariable	*p*-value
	*β*		*β*		*β*		β		*β*		*β*		*β*		*β*	
Age (year)	−0.39 (−0.45, 0.32)	<0.001	0.25 (−0.83, 0.99)	0.866	−0.23 (−0.30, −0.15)	<0.001	−0.10 (−0.19, −0.01)	0.028	−0.51 (−0.61, −0.42)	<0.001	−0.33 (−0.45, −0.22)	<0.001	−0.55 (−0.71, −0.38)	<0.001	−0.26 (−0.46, −0.05)	0.013
BMI (kg/m^2^)	−0.31 (−0.49, −0.13)	<0.001	−0.18 (−0.32, −0.04)	0.014	−0.24 (−0.42, −0.07)	0.006	−0.14 (−0.30, 0.02)	0.088	−0.52 (−0.77, −0.27)	<0.001	−0.35 (−0.56, −0.14)	0.001	−0.64 (−1.04, −0.24)	0.002	−0.43 (−0.80, −0.06)	0.024
Sex	0.13 (−1.02, 1.29)	0.823			1.20 (0.08, 2.32)	0.036	1.15 (0.12, 2.18)	0.029	−0.58 (−2.23, 1.08)	0.494			0.63 (−1.97, 3.24)	0.634		
SBP (mmHg)	−0.13 (−0.16, −0.11)	<0.001	−0.06 (−0.09, −0.03)	<0.001	−0.10 (−0.12, −0.07)	<0.001	−0.05 (−0.09, −0.02)	0.004	−0.18 (−0.21, −0.14)	<0.001	−0.07 (−0.11, −0.02)	0.006	−0.21 (−0.27, −0.16)	<0.001	−0.11 (−0.20, −0.03)	0.007
DBP (mmHg)	−0.16 (−0.20, −0.12)	<0.001	−0.05 (−0.09, −0.00)	0.036	−0.12 (−0.17, −0.08)	<0.001	−0.05 (−0.10, 0.00)	0.066	−0.21 (−0.27, −0.15)	<0.001	−0.06 (−0.13, 0.00)	0.066	−0.26 (−0.36, −0.16)	<0.001	−0.06 (−0.17, 0.06)	0.349
Heart rate (beats/min)	−0.11 (−0.15, −0.06)	<0.001	−0.05 (−0.09, −0.01)	0.021	−0.07 (−0.12, −0.02)	0.004	−0.03 (−0.08, 0.01)	0.154	−0.16 (−0.23, −0.09)	<0.001	−0.08 (−0.14, −0.02)	0.008	−0.25 (−0.36, −0.15)	<0.001	−0.17 (−0.28, −0.07)	0.001
ACEI/ARB	0.26 (−0.66, 1.17)	0.557			0.78 (−0.19, 1.74)	0.114			0.54 (−0.80, 1.88)	0.426			−1.46 (−3.99, 1.07)	0.256		

BMI, body mass index; SBP, systolic blood pressure; DBP, diastolic blood pressure; ACEI, angiotensin converting enzyme inhibitor; ARB, angiotensin II receptor blocker; GLS, global longitudinal strain; GCS, global circumferential strain; GAS, global area strain; GRS, global radial strain.

Values are unstandardized estimate coeffcients (B) and 95% confdent interval (CI).

**Table 5:** Associations between T2DM and the LV strains among hypertensive patients in multivariable analysis.

	GLS		GCS		GAS		GRS	
	Coefficient (95% CI)	*p* value	Coefficient (95% CI)	*p* value	Coefficient (95% CI)	*p* value	Coefficient (95% CI)	*p* value
Model1	0.96 (0.90, 1.03)	0.226	0.94 (0.88, 1.01)	0.076	0.95 (0.91, 1.00)	0.031	1.03 (1.01, 1.06)	0.014
Model2	0.96 (0.90, 1.03)	0.222	0.94 (0.88, 1.01)	0.070	0.95 (0.90, 1.00)	0.029	1.03 (1.01, 1.06)	0.014

GLS, global longitudinal strain; GCS, global circumferential strain; GAS, global area strain; GRS, global radial strain.

Model 1. adjusted for BMI, age, duration of hypertension, SBP, DBP, sex, and heart rate.

Model 2. adjusted for BMI, age, duration of hypertension, SBP, DBP, sex, heart rate and ACEI/ARB use.

Values are unstandardized estimate coeffcients (B) and 95% confdent interval (CI).

## 12 Months later findings in hypertensive patients with diabetes

Univariate linear mixed-model analysis demonstrated a notable decrease in LV strains, specifically LV GCS (*β* = 1.20, 95% CI: 0.38-2.01, *p* = 0.004) and GRS (*β* = −2.82, 95% CI: −4.97–0.68, *p* = 0.010), at the 12-month follow-up. The findings indicated a significant longitudinal association between T2DM and LV strains (see Table 6).

**Table 6:** Changes in 3D-STI-derived parameters during the 12-months follow-up in patients co-occurrence hypertension and diabetes.

	Mean changes between baseline and 12 months	*p* value
LVM	−2.05 (−8.43, 4.33)	0.528
LVMi (g/m^2^)	−0.83 (−4.31, 2.65)	0.639
Interventricular septal thickness (mm)	−0.13 (−0.29, 0.03)	0.114
LVEF (%)	−0.10 (−0.92, 0.73)	0.820
E/A ratio	−0.03 (−0.07, 0.02)	0.215
e’ velocity (cm/s)	−0.38 (−0.77, 0.00)	0.052
E/e’ ratio	0.55 (−0.08, 1.17)	0.084
LV GLS (%)	0.30 (−0.45, 1.06)	0.431
LV GCS (%)	1.20 (0.38, 2.01)	0.004
LV GAS (%)	1.29 (0.09, 2.49)	0.036
LV GRS (%)	−2.82 (−4.97, −0.68)	0.010
Spehericity index	0.01 (−0.01, 0.03)	0.461

LVM, left ventricular mass; LVMi, left ventricular mass index; LVEF, left ventricular ejection fraction; A, transmitral A velocity; E, transmitral E velocity; e’, e’ velocity of mitral annulus; GLS, global longitudinal strain; GCS, global circumferential strain; GAS, global area strain; GRS, global radial strain; EDmass, end-diastolic mass index.

Values in parentheses are 95% CI. Analysis was performed using linear mixed models.

## Discussion

Our study aimed to assess the impact of diabetes on LV structure and function in hypertensive patients using 3D-STI. The results, adjusted for BMI, age, sex, duration of hypertension, SBP, DBP, and heart rate, showed that patients with both diabetes and hypertension exhibited an increased E/e' ratio, and decreased LV GCS, LV GAS, and LV GRS compared to those without diabetes. Furthermore, multivariable analysis indicated that diabetes may influence LV GAS and GRS in hypertensive patients. Assessment after 12 months revealed a deterioration in cardiac indices (LV GCS and GRS) in hypertensive patients.

### The defining features of structural and functional cardiac impairment in individuals with hypertension

Recent studies have utilized two-dimensional speckle tracking echocardiography to assess various components of myocardial deformation in hypertensive patients. This technique has highlighted longitudinal strain as the initial component of systolic deformation to be altered in untreated hypertensive patients (15). These changes are particularly noticeable in the presence of LV hypertrophy (16). The modification in longitudinal strain in hypertensive patients has been linked to LV diastolic abnormalities, potentially attributed to variations in collagen turnover and the emergence of myocardial fibrosis (17). Technological advancements in 3D-STI have led to the development of software that tracks speckle motion regardless of direction, enabling a homogeneous spatial distribution of all three components of the myocardial displacement vector (10). This innovative approach confirms the increase in LV mass detectable by standard echocardiography and enables reliable detection of early myocardial deformation abnormalities (7). Specifically, these abnormalities involve GLS, GRS, and GAS, even in hypertensive patients without LV hypertrophy, exhibiting only minor changes in LV geometry and normal ejection fraction (7). However, the reduction in LV GCS presents a discrepancy. Previous studies have shown that in hypertensive patients, GLS is impaired while GCS remains preserved or compensatorily improved (7, 18). Hypertension can lead to left ventricular hypertrophy and interstitial fibrosis, resulting in longitudinal functional impairment represented by GLS (19–21). However, our findings suggest a more comprehensive impairment of myocardial function, with both GLS and GCS deteriorating concurrently. This phenomenon could be attributed to several underlying mechanisms. First, the progressive hypertrophic and fibrotic remodeling of the left ventricle associated with chronic hypertension may directly compromise both longitudinal and circumferential myocardial contractility, resulting in a concurrent decline in both strain parameters (22–24). Second, prolonged or poorly controlled hypertension may overwhelm the heart's compensatory mechanisms, particularly in patients with advanced or long-standing disease, where the expected compensatory enhancement of GCS fails to occur (25–27). Nevertheless, prior studies did not utilize 3D-STI to conduct stratified analyses to ascertain if the associations of diabetes with changes in LV structure and function are independent of hypertension.

### The structural and functional alterations of the LV in individuals with co-occurring diabetes and hypertension

Around half of patients with hypertension experience insulin resistance, a disturbance in insulin metabolism increasingly linked to hypertension and related cardiovascular diseases (4). Recent analysis of data by Yang et al. demonstrated that individuals diagnosed with both hypertension and diabetes face a higher risk of cardiovascular disease events compared to those with hypertension alone (18). The cardiomyopathy associated with T2DM and hypertension is characterized by pathological changes in the left ventricular myocardium, initially manifesting as diastolic dysfunction with preserved systolic function (4, 28, 29). With disease progression, there is a risk of developing left ventricular systolic dysfunction, which can lead to heart failure with reduced ejection fraction and, in severe cases, mortality (30). A study utilizing cardiac magnetic resonance (CMR) tissue tracking revealed that in patients with hypertension and coexisting T2DM, global and regional strains of the right ventricle were reduced compared to those without diabetes. This suggests that the presence of T2DM exacerbates right ventricular systolic dysfunction in hypertensive patients (18).

Herein, we found that hypertensive patients with diabetes exhibited significantly elevated cardiac functional indices, including the E/e' ratio. Previous research has demonstrated that in individuals with type 2 diabetes, the E/e' ratio provides independent and incremental prognostic information for predicting cardiovascular morbidity and mortality (31). Therefore, the increased E/e' ratio can serve as a valuable indicator of elevated LV filling pressure in diabetes, providing insights into the severity of diastolic dysfunction or heart failure, and potentially indicating the presence of diabetic cardiomyopathy (32). After accounting for other risk factors, diabetes was identified as an independent factor contributing to decreased LV GAS and GRS in hypertensive patients. These findings suggest that the coexistence of diabetes may exacerbate the reduction in LV global strain and the progression of LV remodeling in hypertensive patients. Prior studies have indicated that LV remodeling in hypertension is characterized by distinct cardiac changes, including progressive LV hypertrophy, fibrosis, and myocardial edema, which typically precede diastolic LV dysfunction and are associated with a poorer prognosis (33–37). The deterioration of LV booster strain in individuals with diabetes may result from a combination of more severe metabolic abnormalities, neurohumoral influences, and an increased inflammatory response and immune cell trafficking, all of which contribute to myocardial fibrosis (18, 38–42). Our study provides new insights into the altered LV strain patterns in this population.

Furthermore, our study found that hypertensive patients with diabetes exhibit a significant reduction in LV GRS, while GLS remains largely unchanged, indicating a differential impact of diabetes on myocardial function. The reduction in GRS suggests that diabetes significantly impairs the radial thickening of the myocardium, likely due to metabolic and structural changes such as fibrosis or microvascular damage, which compromise overall myocardial contractility (43–47). Conversely, the stable GLS implies that the longitudinal function of subendocardial fibers may be less affected or more resilient to the combined effects of hypertension and diabetes, potentially due to the preservation of subendocardial fiber function until more advanced myocardial damage occurs (48, 49). This highlights the importance of GRS as a more sensitive marker for detecting myocardial impairment in diabetic hypertensive patients, suggesting that multiple strain parameters should be used for a comprehensive assessment of myocardial function in this population.

Another important and novel finding of this study is the evidence of impaired GAS in patients with hypertension and coexisting T2DM. GAS, derived from 3D-STI, represents the percentage change in myocardial dimensions from their original size, enabling a relatively operator-independent, quantitative assessment of both global and regional LV function (7). Regional strain has been shown to outperform visual assessments in detecting regional wall motion abnormalities and correlates strongly with ejection fraction and the wall motion score index (50–52). Furthermore, GAS can identify mechanical dyssynchrony and respond to cardiac resynchronization therapy (53). A reduction in GAS may be associated with LV remodeling, a common occurrence in patients with hypertension and diabetes, contributing to a decline in overall cardiac function. Importantly, a decrease in GAS can serve as an early marker of subclinical LV dysfunction. In the absence of overt clinical symptoms, changes in GAS can facilitate the early detection of potential myocardial damage, providing an opportunity for timely clinical intervention.

In our study of hypertensive patients, those with concurrent diabetes exhibited decreased LV global strains. Diabetes was found to independently contribute to the reduction in LV GAS and GRS, indicating that T2DM exacerbates the decrease in LV strains in hypertensive patients. These findings underscore the significance of managing blood glucose levels in individuals with hypertension, as diabetes emerges as a significant predictor of alterations in cardiac structure and function.

## Strengths and limitations

The study's primary strengths lie in its utilization of novel echocardiographic modalities to characterize LV function and accurately assess changes in LV mass. Additionally, the longitudinal cardiac monitoring of patients with concurrent hypertension and diabetes, employing linear mixed models to track cardiac remodeling at baseline and after 12 months, enhances the stud's robustness and ability to capture dynamic changes over time.

This study has several limitations. Firstly, due to the relatively small sample size and potential selection bias, the generalizability of our findings may be limited. Future multicenter studies with larger and more diverse samples are needed to validate our results and improve their applicability. Secondly, the impact of hypertension on left atrial structure and function was not assessed, preventing the evaluation of potential increases in left ventricular preload and its effects on LV function, which requires further investigation. Thirdly, we did not account for the differences in glycemic control among diabetic patients, specifically regarding the quality of glycemic control (i.e., HbA1c levels). Although diabetes status was considered, the varying levels of HbA1c may have influenced the left ventricular strain values and clinical outcomes, as suggested by previous studies (54). The lack of a more detailed analysis of HbA1c levels and its correlation with cardiovascular outcomes in this population limits the comprehensiveness of our findings. We recommend future studies include a finer stratification of HbA1c levels to better understand the role of glycemic control in heart failure patients with comorbidities. Additionally, while we adjusted for the use of hypoglycemic treatments, differences in the effects of various treatments (e.g., insulin vs. oral medications) on HbA1c and cardiovascular outcomes were not considered in our analysis, which could be another confounding factor. Fourthly, the study did not account for the age difference between the hypertensive patient group and the healthy control group. Lastly, the lack of animal studies and the investigation of relevant pathological mechanisms in future research is another limitation that should be addressed to improve our understanding of the underlying mechanisms of hypertension-related left ventricular dysfunction.

## Data Availability

The datasets presented in this article will be made available by the authors without undue reservation. Any further enquiries can be directed to yangqm222@163.com.
